# Conjugative Transfer of the pVA1-Type Plasmid Carrying the *pirAB*^*vp*^ Genes Results in the Formation of New AHPND-Causing *Vibrio*

**DOI:** 10.3389/fcimb.2019.00195

**Published:** 2019-06-07

**Authors:** Xuan Dong, Jipeng Song, Jiayuan Chen, Dexi Bi, Wenchao Wang, Yanbei Ren, Hailiang Wang, Guohao Wang, Kathy F. J. Tang, Xuepeng Wang, Jie Huang

**Affiliations:** ^1^Yellow Sea Fisheries Research Institute, Chinese Academy of Fishery Sciences, Laboratory for Marine Fisheries Science and Food Production Processes, National Laboratory for Marine Science and Technology (Qingdao), Key Laboratory of Maricultural Organism Disease Control, Ministry of Agriculture and Rural Affairs, Qingdao Key Laboratory of Mariculture Epidemiology and Biosecurity, Qingdao, China; ^2^Shandong Provincial Key Laboratory of Animal Biotechnology and Disease Control and Prevention, Shandong Agricultural University, Tai'an, China; ^3^Department of Pathology, Shanghai Tenth People's Hospital, Tongji University School of Medicine, Shanghai, China

**Keywords:** acute hepatopancreatic necrosis disease (AHPND), conjugative transfer, pVA1-type plasmid, *pirAB*^*vp*^ genes, bacterial diversity, AHPND-causing *Vibrio* bacteria

## Abstract

Acute hepatopancreatic necrosis disease (AHPND) has caused sharp declines in aquaculture industries of whiteleg shrimp *Penaeus vannamei* in Asia and the Americas since 2010. *Vibrio parahaemolyticus, V. campbellii, V. owensii*, and *V. punensis* have been proved to cause AHPND. However, the mechanisms underlying the burgeoning number of *Vibrio* species that cause AHPND is not known. All of AHPND-causing *Vibrio* bacteria (*V*_AHPND_) harbor a highly homologous plasmid (designated as pVA1-type) carrying *pirAB*^*vp*^ toxin genes. In this study, we demonstrate conclusively that the pVA1-type plasmid can be transferred from *V*_AHPND_ to non-pathogenic bacteria. We constructed a pVPGX1-*Cm*^*r*^ plasmid (a pVA1-type plasmid) by adding a chloramphenicol resistance gene as a marker in a donor AHPND-causing *V. parahaemolyticus* 20130629002S01 (*Vp*2S01). Horizontal transfer of this plasmid was successfully performed from the AHPND-*Vp*2S01 to a non-pathogenic strain of *V. campbellii* at the transfer efficiency of 2.6×10^−8^ transconjugant/recipient, and DNase I treatment did not eliminate the transfer. The recipient *V. campbellii* acquired the pVA1-type plasmid and was shown to produce *pirAB*^*vp*^ RNA and proteins. Challenge studies using the transconjugant caused 100% mortality in exposed groups of *P. vannamei*. The challenged shrimp, infected with the transconjugant bacteria, showed typical gross signs and histological lesions of AHPND. These results demonstrated the conjugative transfer of an AHPND pVA1-type plasmid. It provides timely information for explaining the increased species of AHPND-causing *Vibrio* bacteria and will be useful in the development of management strategies leading to the prevention and control of AHPND.

## Introduction

Acute hepatopancreatic necrosis disease (AHPND, also known as early mortality syndrome, EMS) affects marine shrimp *Penaeus vannamei* and *P. monodon*. Outbreaks of AHPND have been reported in shrimp farms in Asia and the Americas (Zhang et al., 2012; Tran et al., 2013; Gomez-Gil et al., 2014; Gomez-Jimenez et al., 2014; Kondo et al., 2014; Nunan et al., 2014; de la Pena et al., 2015; Lee et al., 2015), where this disease has resulted in substantial production and economic losses, especially in whiteleg shrimp *P. vannamei*. The disease progresses rapidly, usually within 30–35 days, after stocking postlarvae in growth out ponds. The disease emerged in 2010 and is estimated to cause economic losses of several billions of dollars each year (Shinn et al., 2018). This has provided an incentive to determine the causes of the disease.

The causative agent of AHPND was initially determined to be strains of *Vibrio parahaemolyticus* (Zhang et al., 2012; Tran et al., 2013). The virulent strains were found to contain a ~70 kb plasmid (pVA1) carrying genes *pirAB*^*vp*^ that encode homologs of the *Photorhabdus* insect-related (Pir) toxins (Han et al., 2015; Lee et al., 2015). The role of *PirAB*^*vp*^ in causing AHPND was demonstrated in laboratory studies using deletion and insertion mutants (Lee et al., 2015). Recent studies found that AHPND could be caused by strains of other *Vibrio* species, such as *V. harveyi-*like, *V. campbellii, V. owensii*, and *V. punensis* (Kondo et al., 2015; Liu et al., 2015, 2018; Dong et al., 2017b; Han et al., 2017; Restrepo et al., 2018). Strains of these *Vibrio* species, collectively abbreviated as *V*_AHPND_, that contain the pVA1-type plasmids as well as secreting both PirA^*vp*^ and PirB^*vp*^ proteins, can cause AHPND.

The pVA1 plasmid carries a set of genes related to transfer, suggesting that it has the ability to mobilize and transfer from one *Vibrio* cell to another. This set of genes, including conjugative transfer genes, mobilization genes and *pndA* PSK system, was first found in the pVA1 plasmid of AHPND-causing *V. parahaemolyticus* (*Vp*_AHPND_) strain 3HP (Lee et al., 2015). Later, these genes were found in the pVA1-type plasmids from AHPND-causing strains of *V. parahaemolyticus, V. harveyi-*like (*Vh*_AHPND_), *V. owensii* (*Vo*_AHPND_)*, V. campbellii* (*Vc*_AHPND_), and *V. punensis* (*Vpu*_AHPND_) (Kondo et al., 2015; Liu et al., 2015; Dong et al., 2017a,c; Xiao et al., 2017; Restrepo et al., 2018). We reported a high homology between the pVA1-type plasmids of strains of *Vp*_AHPND_ and *Vc*_AHPND_ isolated from the same AHPND-affected pond (Dong et al., 2017a), providing indirect evidence that strains of related *Vibrio* species can become virulent through the transfer of the pVA1-type plasmids. In association with our recent challenge studies, we found *in vivo* horizontal transfer of a pVA1-type plasmid from *Vc*_AHPND_ to non-AHPND-causing *V. owensii* (Dong et al., 2019).

In this work, we demonstrate conclusively, through conjugation experiments, that pVA1-type plasmid can be transferred from AHPND-*V. parahaemolyticus* to *V. campbellii*. This study provides insight into the genetic processes underlying the diversity found among *V*_AHPND_ bacteria.

## Materials and Methods

### Bacterial Strains, Plasmids and Culture Conditions

Bacterial strains and plasmids used are shown in Table 1. *Vp*2S01 was isolated from AHPND-affected shrimp in Guangxi, China in 2013 (Dong et al., 2017a). *Vp*2S01 consists of two chromosomes (Genbank no. CP020034, CP020035) and two plasmids (CP020036, CP020037). *V. campbellii* LMB29 (*Vc*LMB29) was isolated from a skin ulcer sample of red drum in Shenzhen, China (Liu J. et al., 2017). *Vc*LMB29 consists of two chromosomes (CP019293, CP019294) and four plasmids (CP019295-CP019298). *Vibrio* strains were cultured in marine 2216E (5 g/L tryptone, 1 g/L yeast extract, FePO_4_ 0.01g, 1 L filtered seawater, pH adjusted to 7.6) broth or agar, tryptic soy broth with 2% NaCl (TSB+), or Luria Bertani broth (LB) at 28°C.

**Table 1:** Information of *Vibrio parahaemolyticus* and *Vibrio campbellii* strains and plasmids used in this study.

**Strain or plasmid**	**Characteristics**	**Accession no**.	**Identification no**.
**BACTERIA**			
*V. parahaemolyticus*			
20130629002S01 (*Vp*2S01)	Wild type carrying pVPGX1	CP020034- CP020037	PMID: 29051747
*Vp*2S01-*Cm*^r^	*Vp*2S01, *Cm^r^* inserted in pVPGX1		This study
*V. campbellii*			
20130629003S01 (*Vc*3S01)	Wild type carrying pVCGX1, *Rif*^r^	CP020076- CP020081	PMID: 29051747
*V. campbellii* LMB29 (*Vc*LMB29)	Wild type, *Rif*^r^	CP019293- CP019298	Provided by Prof. Zhe Zhao. PMID: 29109705
*E. coli* β2155	A diaminopimelic acid auxotrophic strain		Lab collection
**PLASMID**			
pVPGX1	Wild type carrying *pirAB^*vp*^*	CP020036	PMID: 29051747
pVPGX1-*Cm*^r^	pVPGX1 derivative with *Cm^r^* inserted		This study
pVCGX1	Wild type carrying *pirAB^*vp*^*	CP020078	PMID: 29051747
pKD3	Expression vector containing *Cm*^r^		Lab collection
pUC19	Cloning vector containing *Amp*^r^		Lab collection
pCVD442	Suicide plasmid containing *Amp^r^*		Lab collection

*Amp*^r^, *ampicillin resistant gene*; *Cm*^r^, *chloramphenicol resistance gene*; *Rif*^r^, *rifampin resistance gene*.

### Antibiotic Susceptibility Test

Antibiotic susceptibility was determined by minimal inhibitory concentration (MIC) using the broth microdilution method (Wiegand et al., 2008). Briefly, two-fold serial dilutions of each antibiotic, including ampicillin, cefazolin, ceftriaxone, chloramphenicol, gentamicin, rifampin, streptomycin, and tetracycline, were added into four 96-well microtiter plates, one row for each antibiotic with up to 10 dilutions. A volume of 90 μl bacterial suspension (10^6^ CFU/mL) of each bacterial culture (i.e., *Vp*2S01, *Vp*2S01-*Cm*^*r*^, *Vc*3S01, or *Vc*LMB29) was inoculated into one of the 96-well plates (column 1–11). After adding the antibiotic solution, the final bacterial concentration was 5 × 10^5^ CFU/mL. A volume of 90 μL of broth was added to the control wells of column 12. All plates were incubated at 28°C for 24 h. Breakpoints were defined by the guidelines (M45-A2, 2010 and M100-S24, 2014) of the Clinical and Laboratory Standards Institute (CLSI)^1^.

### Construction of pVPGX1-*Cm*^r^ and *Vp*2S01-*Cm*^r^

To insert the chloramphenicol resistance gene (*Cm*^r^) to the plasmid pVPGX1 by homologous recombination, we amplified the homologous flanking sequences and the *Cm*^r^ fragment (660 bp) by PCR from pVPGX1 and pKD3 plasmids, respectively, using the primer pairs VpCm-5F/5R, VpCm-3F/3R, and Vp-CmF/R (Table 2). The target fragment was amplified using the primer pair VpCm-5F/3R; the PCR products were digested with *Sal*I and ligated with pUC19. After confirming the expression of chloramphenicol in pUC19 by plating the transformed *E. coli* on LB agar plates containing 100 μg/mL of ampicillin and 17 μg/mL of chloramphenicol, the target fragment was then subcloned into pCVD442. The resulting plasmid pCVD442-*Cm*^r^ was introduced into *E. coli* β2155 by electrotransformation. Then, the *E. coli* β2155 carrying pCVD442-*Cm*^r^ was mated with *Vp*2S01 by conjugation. We validated the insertion of *Cm*^*r*^ in the resulting *Vp*2S01-*Cm*^r^ by PCR and sequencing using the primer pairs VpCm-outF/R and CmSeq-F/R (Table 2).

**Table 2:** The nucleotide sequences of PCR primers used in this study.

**Primer**	**Sequence (5^**′**^-3^**′**^)**	**References**
VpCm-5F	ATAGTCGACGCTAAGCAGTTTGAGTCCGTACTG	This study
VpCm-5R	TGGTTTGGTTCTAGATTAAACACAAAATAGAGATG	
VpCm-3F	CTGTGCAAAATTTAACCTCTAATTGTCGC	This study
VpCm-3R	ATAGTCGACATCGAACCTGTGAGGTTAGTACCTC	
Vp-CmF	CATCTCTATTTTGTGTTTAATCTAGAACCAAACCAGGCGCGCCTACCTGTGAC	This study
Vp-CmR	GCGACAATTAGAGGTTAAATTTTGCACAGGGAATAGGAACTTCATTTAAATGGCGCG	
VpCm-outF	GTGGCTTCGGCTTCATTTTCTCTGAATG	This study
VpCm-outR	GTCTTGTGGAACACTGGTAACAGCAAG	
CmSeq-F	GACGGTGAGCTGGTGATATGGGATAGTG	This study
CmSeq-R	CACTATCCCATATCACCAGCTCACCGTC	
VpPirA-284F	TGACTATTCTCACGATTGGACTG	Han et al., 2015
VpPirA-284R	CACGACTAGCGCCATTGTTA	
VpPirB-392F	TGATGAAGTGATGGGTGCTC	Han et al., 2015
VpPirB-392R	TGTAAGCGCCGTTTAACTCA	
AP1F	CCTTGGGTGTGCTTAGAGGATG	Flegel and Lo, 2014
AP1R	GCAAACTATCGCGCAGAACACC	
AP2F	TCACCCGAATGCTCGCTTGTGG	Flegel and Lo, 2014
AP2R	CGTCGCTACTGTCTAGCTGAAG	
Vp-groelF	AGGTCAGGCTAAGCGCGTAAGC	Hossain et al., 2012
Vp-groelR	GTCACCGTATTCACCCGTCGCT	
Vca-hly5	CTATTGGTGGAACGCAC	Haldar et al., 2010
Vca-hly3	GTATTCTGTCCATACAAAC	

*GTCGAC: recognition sequence for Sal1*.

### Conjugation Experiments

Conjugation experiments were carried out using a protocol described by Wiesner et al. (2013). Briefly, *Vp*2S01-*Cm*^r^ (donor) was cultured in 2216E with chloramphenicol (Cm, 20 μg/mL); *Vc*LMB29 (recipient) was cultured in 2216E with rifampin (Rif, 15 μg/mL). Equal amounts (50 mL each) of secondary inocula of donor and recipient cells were mixed, then washed and resuspended twice with 4 mL fresh 2216E to remove residual antibiotics. The mixture was resuspended with 100 μL fresh 2216E and dropped on 0.22 μm Millipore filter membranes placed on a 2216E agar plate and incubated at 28°C for 12 h. The conjugation mix was then resuspended in 2 mL of 2216E broth, diluted to 10^−1^, 10^−2^, 10^−3^, and spread on 2216E plates which contained Cm (60 μg/mL) and Rif (20 μg/mL) as transconjugants selection antibiotics. The mixture was further diluted to 10^−7^, 10^−8^, 10^−9^, then spread on 2216E plates containing Rif for determining the number of recipient cells. Transfer efficiency was determined by the ratio of transconjugants' counts to those of recipient strain. Transconjugants were analyzed for the presence of *pirAB*^*vp*^ genes and backbone of pVA1 plasmid with 4 pairs of PCR primers: VpPirA-284F/R, VpPirB-392F/R, AP1F/R, AP2F/R (Table 2). VpPirA-284F/R and VpPirB-392F/R primers target *pirA*^*vp*^ and *pirB*^*vp*^ gene, respectively (Han et al., 2015). AP1F/R and AP2F/R primers targeted the backbone of pVA1 plasmid (Flegel and Lo, 2014).

To determine which of the bacterial colonies grown on Cm and Rif plates were *V. campbellii*, all were analyzed with 2 pairs of PCR primers Vca-hly5/3 (amplicon length: 328 bp) and Vp-groelF/R (510 bp) (Table 2), which can be used to distinguish *V. parahaemolyticus* and *V. campbellii* (Haldar et al., 2010; Hossain et al., 2012).

In order to study the stability of pVA1-type plasmid in transconjugants, one transconjugant colony (named as *Vc*LMB29-pVPGX1) was passed 5 times on 2216E plates which containing Cm (60 μg/mL) and Rif (20 μg/mL) and analyzed for the presence of *pirAB*^*vp*^ genes with 4 pairs of PCR primers: VpPirA-284F/R VpPirB-392F/R, AP1F/R, and AP2F/R (Table 2).

### Conjugation Experiments With DNase I

To determine the effect of DNase in plasmid transfer, this enzyme was added to mating mixture in conjugation experiments. The conjugation were carried out as described above. 2.5 μL of DNase I (5 unit; NEB) was added to the mixture of donor and recipient cells and washed with 4 mL 2216E. The mixture was resuspended with 90 μL fresh 2216E and 10 μL of DNase I (20 unit; NEB), then, dropped on a 0.22 μm Millipore filter membrane placed on the 2216E agar plate and incubated at 28°C for 12 h. The transfer efficiency was determined as described above.

### Expression of *pirAB^*vp*^* Genes in pVA1-Type Plasmid From a Transconjugant

For determination of the expression of *pirAB*^*vp*^ genes, total RNA of *Vc*LMB29-pVPGX1, *Vc*LMB29, and *Vp*2S01-*Cm*^r^ were extracted using RNAprep pure Cell/Bacteria Kit (TIANGEN Biotech, China) and treated with DNase I. RT-PCR were performed to analyze the mRNA expression of *pirA*^*vp*^ and *pirB*^*vp*^ genes using 2 pairs of PCR primers VpPirA-284F/R and VpPirB-392F/R. The cDNA was generated with Random 6 mers at 42°C for 45 min, 95°C for 5 min using the TaKaRa PrimeScript^TM^ II 1st Strand cDNA Synthesis Kit (Takara Bio, Japan). The PCR were initiated at 94°C for 7 min, followed by 35 cycles of 94°C for 30 s, 60°C for 30 s, and 72°C for 30 s, ending with 72°C for 7 min. Following PCR, 5 μL of the reaction mixture was analyzed in a 2% agarose gel containing GeneFinder (Bio-V, China).

To determine the production of PirA^*vp*^ and PirB^*vp*^ in the transconjugant, individual colonies of *Vc*LMB29-pVPGX1, *Vc*LMB29 (the recipient non-AHPND strain as a negative control), and *Vp*2S01 (an *Vp*_AHPND_ strain as a positive control) were cultured in 2216E broth at 28°C for 10 h. Fifty milliliter of each culture was centrifuged at 4,280 g for 10 min, each supernatant was concentrated into 2 mL by using Amicon 10 kDa filter tubes. All samples were analyzed using 12% sodium dodecyl sulfate-polyacrylamide gel electrophoresis (SDS-PAGE) (Laemmli, 1970) and then proteins in the target bands were identified by mass spectrometry (MS) analysis (Wang et al., 2011). All data were integrated and analyzed by the use of GPS Explorer V3.6 software with the MASCOT V2.3 search engine (Matrix Science Ltd., UK) with the following parameters: NCBI nr, trypsin digest with one missing cleavage, MS tolerance at 200 ppm, and MS/MS tolerance of 0.8 Da. Proteins were identified based on 95% or higher confidence interval of their scores.

### Sequencing and Assembly of pVA1-type Plasmid Sequence From the Transconjugant

The sequence of pVCON1 from *Vc*LMB29-pVPGX1 was determined after sequencing the bacteria with an Illumina HiSeq X Ten Sequencer (PE150 mode) and filtering out the bacterial chromosomal sequence (Liu J. et al., 2017). Raw sequencing data was generated by Illumina base calling software CASAVA v1.8.2 (http://support.illumina.com/sequencing/sequencing_software/casava.ilmn). Contamination reads were identified by Trimmomatic (http://www.usadellab.org/cms/uploads/supplementary/Trimmomatic) with default parameters. Clean data obtained by above quality control processes were used for further analysis.

We used ABySS (http://www.bcgsc.ca/platform/bioinfo/software/abyss) to perform a sequence assembly with multiple-Kmer parameters (Jackman et al., 2017). GapCloser software (https://sourceforge.net/projects/soapdenovo2/files/GapCloser/) was subsequently applied to fill up the remaining local inner gaps and correct the single nucleotide polymorphism for the final assembly results.

### Comparative pVA1-type Plasmid Sequences Analysis

The reference sequences of pVA1-type plasmids downloaded from NCBI were used for comparative sequences analysis. These sequences included pVA1 (KP324996), pVPGX1 (CP020036), pVCGX1 (CP020078), and pLA16-2 (CP021148). We used the BLAST Ring Image Generator (BRIG) to generate the circle map. The innermost ring shows the reference sequence of pVA1. The second innermost ring shows the sequence of pVPGX1.The third innermost ring shows the sequence of pVCON1. The forth innermost ring shows the sequence of pVCGX1. The fifth innermost ring shows the sequence of pLA16-2. The sixth innermost ring shows the ORFs of the forward chain of pVA1. The seventh innermost ring shows the ORFs of the complementary chain of pVA1. The outermost ring highlights the gene clusters of pVA1.

### Challenge Bioassays and *pirAB^*vp*^* RT-PCR

To determine AHPND pathogenicity of the transconjugant *Vc*LMB29-pVPGX1, a challenge study was undertaken. We used three *Vibrio* strains in the study, including *Vp*2S01-*Cm*^r^ as the positive control, *Vc*LMB29-pVPGX1 as the target strain, and *Vc*LMB29 as the negative control. A blank control without *Vibrio* bacteria was also included. Each *Vibrio* strains was cultured in TSB+ at 28°C until its OD_600nm_ reached 0.8–0.9 (~8–12 h) and added into 4 L seawater to make bacterial suspension at a concentration of 1 × 10^8^ CFU/mL. Each group of 45–56 healthy *P. vannamei* (mean weight: 1 g) were immersed in each of the bacterial suspensions for 15 min. For each group, the shrimp and bacterial suspension were then divided into three parallels of 30 L tanks filled with seawater to give a final bacterial concentration of 1 × 10^6^ CFU/mL. The shrimp in the blank control group were immersed in marine 2216E broth. The mortality of shrimp in each tank was monitored and recorded every 8 h. Moribund shrimp were fixed for histopathological examination with Davidson's alcohol-formalin-acetic acid (Bell and Lightner, 1988).

In order to analyze the expression of *pirAB*^*vp*^ toxin genes in shrimp, tissues of hepatopancreas and stomach from moribund shrimp were sampled to extract total RNA using RNAprep Pure Tissue Kit (TIANGEN Biotech, China) along with treatment with 0.3 U/μL DNase I at 25°C for 15 min. RT-PCR were performed to determine the mRNA expression of *pirAB*^*vp*^ genes as described above.

### Nucleotide Sequence Accession Number

Complete plasmid sequence of pVCON1 from strain *Vc*LMB29-pVPGX1 has been deposited in GenBank under the accession MH890610.

## Results

### Comparative Sequence Analysis of pVA1-type Plasmids in AHPND-Causing *Vibrio* Strains

**Figure 1:**
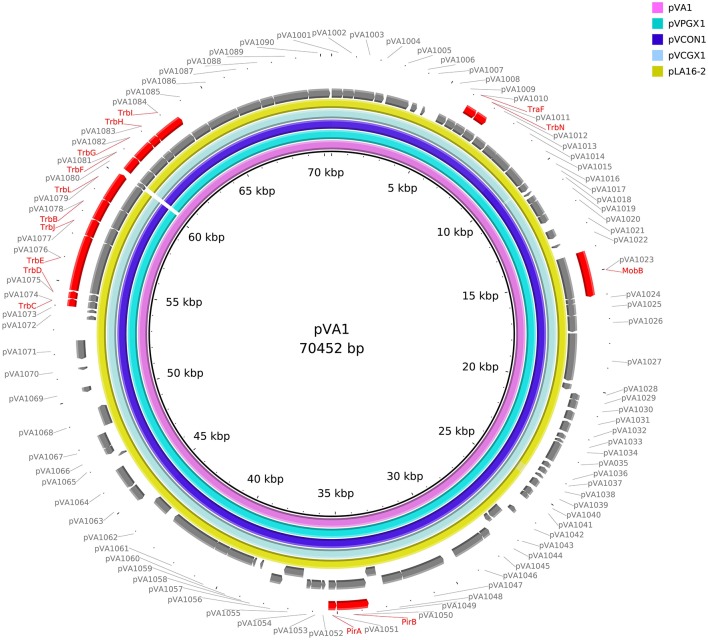
Comparative sequences analysis of pVA1-type plasmids. The reference sequences of pVA1-type plasmids downloaded from NCBI were used for comparative plasmid sequences analysis. The tracks from inside to outside represent the pVA1-type plasmid sequences of pVA1 (KP324996), pVPGX1 (CP020036), pVCON1 (MH890610), pVCGX1 (CP020078), pLA16-2 (CP021148), ORF (+) of pVA1, ORF (–) of pVA1, and marked genes, ORF numbers.

We retrieved 23, complete or draft, sequences of pVA1-type plasmids harbored in *V*_AHPND_ bacteria from the NCBI RefSeq database (Table 3). There were 9 completely sequenced pVA1 plasmids, ranging in size from 69 to 73 kb. These plasmids have 99.92–100.00% identity in nucleotide sequence. We compared, with regard to genes known to be involved in conjugation, the genes of these plasmids, and found that all of the plasmid genomes contained two gene clusters related to conjugation (Figure 1). In one 9.5 kb gene cluster, we found a T4SS gene cluster with 11 genes (CDSs: pVA1074 to PVA1084, based on the reference plasmid pVA1) that, with the exception of pVA1080, code for conjugative transfer genes (trbC, trbD, trbF, trbJ, trbB, trbL, trbF, trbG, trbH, trbI). In the second gene cluster, pVA1010 and pVA1011 were found to be related to conjugation, the CDSs of which have high homology to T4SS-related proteins TraF and TrbN. The pVA1023 is a conjugative gene predicted to encode molybdopterin-guanine dinucleotide biosynthesis protein MobB (Figure 1).

**Table 3:** pVA1-type plasmids harbored in AHPND-causing *Vibrio* strains.

**Number**	**Strains name**	**Species**	**Sample source**	**Collected date**	**Country**	**References**
1	KC13.17.5	*V. h*.-like	*P. vannamei*	2013	Vietnam	PMID: 26383659
2	NCKU_CV_CHN	*V. p*.	Shrimp	2010	China	PMID: 25189578
3	NCKU_TV_5HP	*V. p*.	*P. vannamei*	2012	Thailand	PMID: 25189578
4	M0605	*V. p*.	*P. vannamei*	2013	Mexico	PMID: 24604636
5	TUMSAT_D06_S3	*V. p*.	Shrimp	Unkn	Thailand	PMID: 24723705
6	TUMSAT_DE2_S2	*V. p*.	Shrimp	Unkn	Thailand	PMID: 24723705
7	TUMSAT_DE1_S1	*V. p*.	Shrimp	Unkn	Thailand	PMID: 24723705
8	Ba94C2	*V. p*.	Shrimp	2015	S. A.	PMID: 27570736
9	SH14	*V. o*.	*P. vannamei*	2013	China	PMID: 26634753
10	13-028/A3	*V. p*.	penaeid shrimp	2013	Vietnam	PMID: 25667334
11	NCKU_TV_3HP	*V. p*.	*P. vannamei*	2012	Thailand	PMID: 25189578
12	VPE61a	*V. p*.	Shrimp	2013	Thailand	PMID: 28404482
13	LA16-V1	*V. c*.	*P. vannamei*	2016	Ecuador	PMID: 28912332
14	20130629003S01	*V. c*.	*P. vannamei*	2013	China	PMID: 29051747
15	20130629002S01	*V. p*.	*P. vannamei*	2013	China	PMID: 29051747
16	1930	*V. p*.	*P. vannamei*	2014	China	PMID: 29234316
17	HZ-7	*V. p*.	*P. vannamei*	2016	China	PMID: 29234316
18	MVP1	*V. p*.	Shrimp	2016	Malaysia	PMID: 29234316
19	MVP2	*V. p*.	Shrimp	2016	Malaysia	PMID: 29234316
20	MVP6	*V. p*.	Shrimp	2016	Malaysia	PMID: 29234316
21	v110	*V. p*.	Shrimp	2013	China	PMID: 23788537
22	BA55	*V. pu*.	*P. vannamei*	2015	S. A.	PMID: 30166588
23	PB1937	*V. p*.	Shrimp	2012	China	G.A.: CP022245.1

*V. h.-like, Vibrio harveyi-like; V. p., Vibrio parahaemolyticus; V. o., Vibrio owensii; V. c., Vibrio campbellii; V. pu., Vibrio punensis; Unkn, unknown; G.A., GenBank Accession number; S. A., South America*.

### Construction of Chloramphenicol Resistant Strain *Vp*2S01-*Cm*^r^ and Antibiotic Resistance Patterns of *Vibrio* Bacteria

To determine if the pVA1-type plasmid can be horizontally transferred, we constructed a testing plasmid in the donor strain *Vp*2S01-*Cm*^r^ by homologous recombination, incorporating a chloramphenicol resistance gene (*Cm*^r^) into its virulence plasmid pVPGX1 (69,227 bp). The *Cm*^r^ was inserted between the ORF9 (*Vp*2S01_p10009) and ORF10 (*Vp*2S01_p10010) region, the size of pVPGX1-*Cm*^r^ is 70,125 bp.

All 3 *V*_AHPND_ strains (*Vp*2S01, *Vp*2S01-*Cm*^r^, *Vc*3S01) and a non-pathogenic strain (*Vc*LMB29) were determined for their antibiotic resistance. The results of MIC tests showed that, of the four strains tested, only *Vp*2S01 was susceptible to chloramphenicol (Cm) (Table 4). Among the other 7 antibiotics analyzed, this strain was intermediate to both ceftriaxone (Cro) and rifampin (Rif). Strain *Vp*2S01-*Cm*^r^ exhibited a similar pattern of antibiotic resistance to that of *Vp*2S01 except that it was resistant to Cm as expected. Strain *Vc*3S01 was resistant to 7 of the antibiotics and was only susceptible to Cm. The *Vc*LMB29 exhibited intermediate to Cm, but resistant to Rif and susceptible to 6 other antibiotics. Thus, Cm and Rif were selected to screen transconjugants.

**Table 4:** Minimal inhibitory concentrations (MICs) of selected antibiotics on *Vibrio parahaemolyticus* and *V. campbellii*.

**Antibiotics**	**MIC (μg/mL)**				**Interpretive criteria**			**References**
	***Vp*** **2S01**	***Vp*2S01** **-*Cm*^**r**^**	***Vc*** **3S01**	***Vc*** **LMB29**	**S**	**I**	**R**	
Amp	512	>512	512	ND	≤8	16	≥32	(a)
Cfz	128	64	256	ND	≤1	2	≥4	(a)
Cro	2	2	4	ND	≤1	2	≥4	(b)
Cm	4	128	8	16	≤8	16	≥32	(a)
Gen	64	32	64	ND	≤4	8	≥16	(a)
Rif	2	2	64	32	≤1	2	≥4	(b)
Str	128	128	512	ND	–	–	–	(b)
Tet	32	64	64	ND	≤4	8	≥16	(a)

*Abbreviation of antibiotics: Amp, ampicillin; Cfz, cefazolin; Cro, ceftriaxone; Cm, chloramphenicol; Gen, gentamicin; Rif, rifampin; Str, streptomycin; Tet, tetracycline; R, resistant; I, intermediate; S, sensitive; ND, not detected; (a): CLSI M45-A2 (2010)^1^; (b): CLSI M100-S24 (2014)^1^. Antibiotics in the table were selected for comparison of the antibiotic susceptibility of Vp2S01, Vp2S01-Cm^r^, Vc3S01 and VcLMB29, not for clinical use*.

### Conjugation, Stability of pVA1-type Plasmid and Expression of *pirAB^*vp*^* Genes

In conjugation experiments, filter mating protocol was used to transfer the pVpGX1-*Cm*^*r*^ plasmid from *Vp*_AHPND_ strain *Vp*2S01-*Cm*^r^ (donor) to *V. campbellii* strain *Vc*LMB29 (recipient). The transconjugants were obtained by selecting for colonies that were resistant to both Cm and Rif and were further verified to be *V. campbellii* by species-specific PCR showing that a 328 bp fragment was generated with Vca-hly5/3 primers. We found positive transconjugants and the transfer efficiency was 2.6 × 10^−8^ transconjugant/recipient at 12 h of conjugation. A similar transfer efficiency, 6.0 × 10^−9^, was obtained when DNase I was added to the conjugation mixture. One transconjugant *Vc*LMB29-pVPGX1 was passed for 5 times, and all of the subcultures had positive PCR reactions with primers VpPirA-284F/R VpPirB-392F/R, AP1F/R and AP2F/R, indicating that pVA1-type plasmid was stable in this transconjugant.

**Figure 2:**
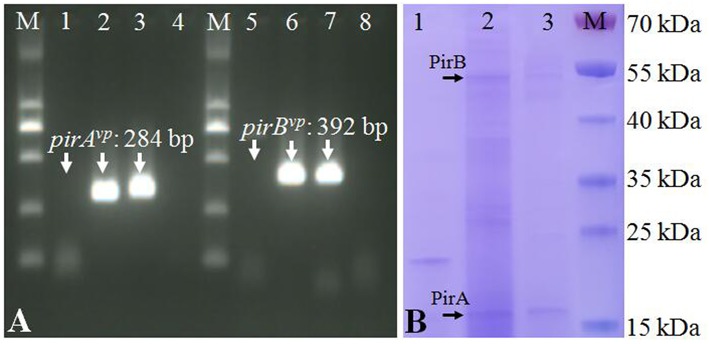
Expression of *pirAB*^*vp*^ genes in *Vibrio campbellii* and *V. parahaemolyticus*. **(A)** RT-PCR detection of *pirA*^*vp*^ and *pirB*^*vp*^ expression. Lanes 1–4: RT-PCR amplification of *pirA*^*vp*^ in RNA samples from strain *Vc*LMB29, *Vc*LMB29-pVPGX1, *Vp*2S01-*Cm*^*r*^, and water (blank control), respectively. Lanes 5–8: RT-PCR amplification of *pirB*^*vp*^ in RNA samples from strain *Vc*LMB29, *Vc*LMB29-pVPGX1, *Vp*2S01-*Cm*^*r*^, and water (blank control), respectively. M: 2 kb plus DNA ladder. **(B)** Sodium dodecyl sulfate-polyacrylamide gel electrophoresis (SDS-PAGE) analysis of PirA^*vp*^ and PirB^*vp*^. Lane 1: *Vc*LMB29. Lane 2: *Vc*LMB29-pVPGX1. Lane 3: *Vp*2S01. M: the PageRuler Prestained Protein Ladder.

The strain *Vc*LMB29-pVPGX1 grown to a mid-logarithmic phase was pelleted. Total RNA was extracted and analyzed for the expression of *pirAB*^*vp*^ using RT-PCR. The result showed that bands of *pirA*^*vp*^ and *pirB*^*vp*^ products appeared at expected sizes of 284 and 392 bp in the transconjugant (Figure 2A, lanes 2 and 6), not seen in the recipient bacteria (Figure 2A, lanes 1 and 5). There were no amplified products when the RT step was omitted. Furthermore, the extracellular extracts from the cultured bacteria were analyzed for the presence of PirA^*vp*^ and PirB^*vp*^ proteins in an SDS-PAGE. The result showed that 2 bands, 17 kDa (predicted size of PirA^*vp*^) and 50 kDa (predicted size of PirB^*vp*^), were observed in the sample from strain *Vc*LMB29-pVPGX1 (Figure 2B, lane 2) and in the *Vp*_AHPND_ strain *Vp*2S01 (as the positive control, Figure 2B, lane 3). The 17 and 50 kDa bands from *Vc*LMB29-pVPGX1 (Figure 2B, lane 2) were excised and analyzed by mass spectrometry (LC-MS/MS) and MASCOT, confirming the presence of PirA^*vp*^ and PirB^*vp*^ proteins. These two bands were not present in the sample of the recipient bacteria *Vc*LMB29 (Figure 2B, lane 1).

### Sequence Analysis of pVA1-type Plasmids From Donor and Transconjugant Cells

To determine if strain *Vc*LMB29-pVPGX1 acquired the pVPGX1-*Cm*^r^, the bacterial genome was sequenced and compared with that of recipient strain VcLMB29. The result showed that the *Vc*LMB29-pVPGX1 contains a large (70,053 bp, named as pVCON1) plasmid. This transconjugant was determined to be the recipient bacterium based on that its chromosomal sequence was 99.9% identical to that of *Vc*LMB29. The pVCON1 displayed a 100% identity to pVPGX1-*Cm*^r^ from donor *Vp*2S01-*Cm*^*r*^. Further comparative sequence analysis among the pVA1-type plasmids, including pVA1, pVPGX1, pVCON1, pVCGX1 and pLA16-2, revealed that pVCON1 sequence shared high (>99%) homologies with those in other pVA1-type plasmid (Figure 1). Interestingly, the smaller plasmid pVPGX2 (38 kb) from *Vp*2S01-*Cm*^r^ was not found in *Vc*LMB29-pVPGX1.

### Ability of *Vc*LMB29-pVPGX1 to cause AHPND in *P. vannamei*

**Figure 3:**
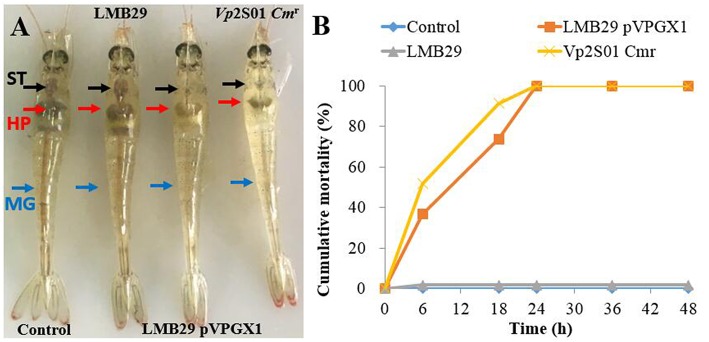
Gross signs and mortality of *Penaeus vannamei* exposed to AHPND-bacteria. **(A)** Gross signs of AHPND-affected shrimp. Normal shrimp in the blank control and the group infected with *Vc*LMB29: a normal size hepatopancreas (HP) with dark orange color and a full stomach (ST) and midgut (MG). AHPND-affected shrimp from the group infected with *Vc*LMB29-pVPGX1 and the group infected with *Vp*2S01-*Cm*^r^: pale, atrophied HP, and an empty stomach (ST) and midgut (MG). **(B)** Cumulative mortality of shrimp infected with *Vc*LMB29-pVPGX1, shrimp were exposed to *Virbio* bacteria through immersion infection.

From the laboratory bioassays, *P. vannamei* exposed to *Vc*LMB29-pVPGX1 or *Vp*2S01-*Cm*^r^ showed typical gross signs of AHPND within 6 h, displaying a pale and atrophied hepatopancreas (HP), and an empty stomach (ST) and midgut (MG) (Figure 3A); all the exposed shrimp died within 24 h (Figure 3B). The shrimp in the negative control and blank control groups had a normal size HP, dark orange in color, and a full ST and MG.

**Figure 4:**
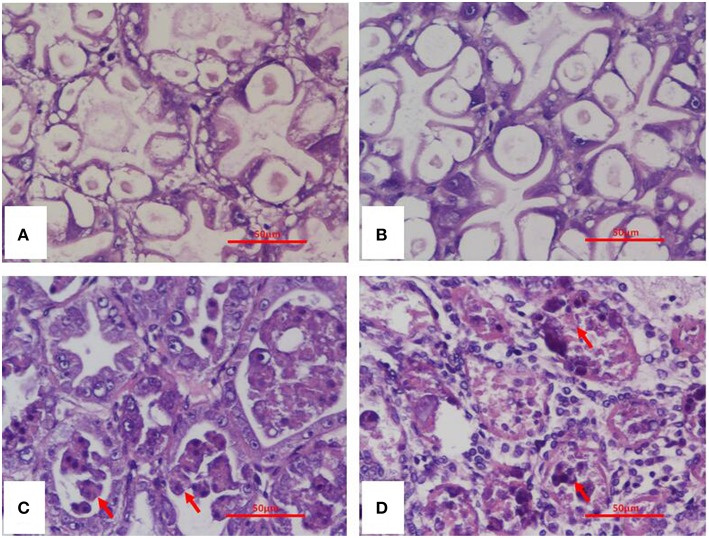
H&E stained histological sections of the hepatopancreas of *Penaeus vannamei* from AHPND-*Vibrio* bacteria challenge studies. **(A)** Healthy (blank-control) shrimp; **(B)** shrimp infected with *Vc*LMB29; **(C)** shrimp infected with *Vc*LMB29-pVPGX1; **(D)** shrimp infected with *Vp*2S01-*Cm*^r^. **(A,B)** normal histology of hepatopancreas; **(C,D)** AHPND histopathology characterized by sloughing epithelial cells of hepatopancreatic tubule (arrows). Scale bars = 50 μm.

**Figure 5:**
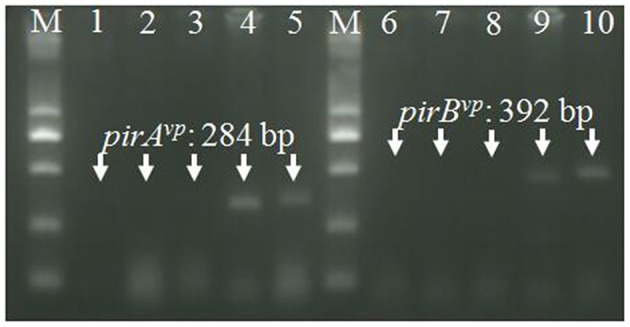
RT-PCR detection of *pirA*^*vp*^ and *pirB*^*vp*^ mRNA. Total RNA extracted from tissues in cephalothorax containing hepatopancreas and stomach were analyzed by RT-PCR using primers VpPirA-284F/R (lanes 1–5) and primers VpPirB-392F/R (lanes 6–10). Lanes 1 and 6: non-template control, lanes 2 and 7: healthy shrimp, lanes 3 and 8: shrimp infected with *Vc*LMB29, lanes 4 and 9: shrimp infected with *Vc*LMB29-pVPGX1; lanes 5 and 10: shrimp infected with *Vp*2S01-*Cm*^r^. M: 2 kb plus DNA ladder.

Histopathological examination of moribund shrimp infected with *Vc*LMB29-pVPGX1 or *Vp*2S01-*Cm*^r^ revealed typical AHPND lesions in the HP compare to that in the negative control and blank control groups (Figures 4A,B). These lesions were characterized by acute sloughing of epithelium cells in the HP tubules, followed by extensive necrosis in most tubules (Figures 4C,D). The PCR detection of *Vibrio* bacteria showed that *V. parahaemolyticus* could be detected in all groups and *V. campbellii* could be only detected in LMB29-infected shrimp and LMB29 pVPGX1-infected shrimp (Supplementary Figure 1). In addition, we investigated the expression of *pirA*^*vp*^ and *pirB*^*vp*^ in shrimp by RT-PCR using primers VpPirA-284F/R and VpPirB-392F/R. The result showed that bands of both amplificated products appeared at expected sizes of 284 bp from *pirA*^*vp*^ and 392 bp from *pirB*^*vp*^ in the sample of the shrimp infected with *Vc*LMB29-pVPGX1 (Figure 5, lanes 4 and 9). There were no amplification products from the healthy shrimp and the shrimp infected with non-AHPND-causing *V. campbellii* strain *Vc*LMB29 (Figure 5, lanes 2, 3 and 6, 7).

## Discussion

Horizontal gene transfer is a driver for diversification of pathogenic bacteria via mobile genetic elements (MGEs) including plasmids, transposons, insertion sequences (ISs), prophages, integrons and associated gene cassettes, integrative conjugative elements (ICEs), and genomic islands (GIs) (Antonenka et al., 2005; Bi, 2015). However, little is known in this regard in relation to pathogenic bacteria of crustaceans. This is the first study to definitively show conjugative transfer of the pVA1-type plasmid which exists in AHPND-*Vibrio* bacteria.

Initially, it was thought that the virulence of *Vp*_AHPND_ may be due to lysogenic infection of the *V. parahaemolyticus* genome with a bacteriophage (FAO, 2013), as lysogenic phages frequently harbor bacterial toxin genes. For example, *V. cholera* toxin is encoded by ctxAB genes residing on the CTXϕ phage (Waldor and Mekalanos, 1996). However, our study have found lysogenic phages in only two of five *Vp*_AHPND_ isolates (Wang et al., 2016), indicating that the AHPND toxin genes do not reside on a phage. Our previous study showed that pVA1-type plasmids from *Vp*_AHPND_ (*Vp*2S01) and *Vc*_AHPND_ (*Vc*3S01), isolated from a diseased pond, had a sequence similarity of 99.9% (Dong et al., 2017a). Then, in a subsequent study, we found that *Vo*_AHPND_ appeared in a tank where shrimp were being challenged with *Vc*_AHPND_ (Dong et al., 2019). Furthermore, pVA1-type plasmids can be found in all published sequences of AHPND-causing *Vibrio* strains (Table 3). All of these results suggested the possible horizontal transfer of pVA1-type plasmids from AHPND-*Vibrio* to non-pathogenic bacteria. In the current study, the transfer of the pVA1-type plasmid from *Vp*2S01-*Cm*^r^ to *V. campbellii* was demonstrated through conjugation experiments.

The demonstration of the transfer of a virulent plasmid to non-pathogenic strains of *Vibrio* has practical relevance to the development of protocols for the diagnosis of AHPND and for strategies for prevention and control of the disease. For example, some shrimp hatcheries provide a quality analysis of postlarvae based on the number of *V. parahaemolyticus* and related bacteria. The procedure involves culturing *Vibrio* bacteria from hepatopancreas tissue homogenates on TCBS agar plates, in these tests, colonies of *V*. *parahaemolyticus* are green. The CFU of a particular sample is usually determined only by the counts of green colonies; however, this would miss virulent strains of other *Vibrio* species present, such as *V. owensii*, potentially reducing the effectiveness of this metric. In addition, some shrimp farms routinely use probiotics, many of which include or are contaminated with species of *Vibrio*, to control the disease and to improve water quality; however, increasing levels of *Vibrio* in the pond water could also increase the risk of the virulent plasmid being transferred and could subsequently increase the risk of AHPND. Also, antibiotics, antimicrobial peptides, and phages have been considered in aquaculture farms and hatcheries to control the bacterial diseases as prophylactic and therapeutic agents (Jun et al., 2016, 2018). If pVA1 transfers to other bacteria in the shrimp or pond environment, the new strains may not be susceptible to these treatments.

This study showed that pVA1-type plasmid could be conjugatively transferred with an efficiency of 10^−8^ in a 12 h period. Thus, such transfer events may be very frequent in diseased shrimp ponds, where numerous *Vibrio* spp. are present. The conjugation process can bring a high risk of developing the diversity and complexity for AHPND bacteria.

There are over 100 species of bacteria in the genus *Vibrio*. Of these, only 4 have been reported to contain pVA1-type plasmids. Three of the four species are closely related and in the *harveyi*-clade, and the fourth, *V. punensis* (Restrepo et al., 2018), is a more distant relative within the genus. This suggests that the number of potential recipient strains, those capable of receiving AHPND toxin genes and attaining virulence through horizontal transfer of a pVA1-type plasmid, is relatively small. This is not unexpected as, in general, the horizontal exchange of genetic material through conjugation occurs only between closely related individuals (Bernstein et al., 2018). Therefore, management of pond microbial ecology to reduce the prevalence of these potential recipient strains, such as through the use of probiotics to effect competitive exclusion, could prove to be a useful strategy to reduce the risks of AHPND. It would be useful to determine the range of bacterial species that are capable of being recipients for the pVA1-type plasmids. This can be studied through the use of established pVA1 plasmid transfer systems, both *in vitro* (described here) and *in vivo* (Dong et al., 2019). Recent studies have shown that the conjugation efficiency is affected by both duration and temperature (Wiesner et al., 2013; Liu y. et al., 2017). In order to effectively assess the risk, more information is needed on factors affecting transfer efficiency of pVA1-type plasmids in pond environments.

## Data Availability

The datasets generated for this study can be found in Complete plasmid sequence of pVCON1 from strain VcLMB29-pVPGX1 has been deposited in GenBank under the accession MH890610.

## Author Contributions

The experiments, data analysis, and manuscript writing were performed by JS, XD, and JH. HW isolated and identified the bacterial strain 20130629003S01 and provide the *Vibrio campbellii* strain 20130629003S01, *V. parahaemolyticus* strain 20130629002S01 and revised the manuscript. JS, JC, WW, GW, and YR performed experiments. XW and KT discussed the results and revised the manuscript. DB provided vital guidance, technical support, and proofreading for the work. All authors read and approved the final manuscript.

### Conflict of Interest Statement

The authors declare that the research was conducted in the absence of any commercial or financial relationships that could be construed as a potential conflict of interest.

## Footnotes

^1^https://clsi.org/standards/products/microbiology/documents
